# A Novel Mutation of Beta-ketothiolase Deficiency: The First Report from Iran and Review of Literature

**Published:** 2018

**Authors:** Rahim VAKILI, Somayyeh HASHEMIAN

**Affiliations:** 1 Department of Pediatric Endocrinology and Metabolism, Imam Reza Hospital, Faculty of Medicine, Mashhad University of Medical Sciences, Mashhad, Iran; 2 Medical Genetic Research Center, Mashhad University of Medical Sciences, Mashhad, Iran

**Keywords:** Beta-ketothiolase deficiency, encephalopathy like symptoms, genetic assay

## Abstract

Beta-ketothiolase deficiency is a rare autosomal recessive disorder characterized by an inborn error of isoleucine catabolism and affecting ketone body metabolism. Clinical features characterized by intermittent keto acidotic episodes are associated with clinical signs and symptoms of toxic encephalopathy such as lethargy, hypotonia, vomiting, tachypnea, and coma in some patients, with an onset during infancy or toddler-hood. A two months old girl presented to pediatric ward of Imam Reza Hospital in Mashhad City, Northwestern Iran in October 2016, with acute episode of fever and toxic encephalopathy with attack of vomiting, hypotonia, lethargy, tonic-clonic seizures and then a day in coma, few days after vaccination. After then similar episodes happened until 7 months age. Bio chemical tests that suggested diagnose of beta ketothiolase deficiency were attacks of ketoacidosis with urinary exertion of 2-methyl-3-hydroxybutyric acid 2-methyl aceto acetic acid tiglylglycine. In genetic assessment, we detected a novel homozygous mutation c.664A> C (p. Ser 222 Arg) in ACAT gene. This is the first report of beta ketothiolase deficiency confirmed by molecular analysis from Iran. We report on a homozygous variant in the ACAT1 gene and that is a novel mutation. We recommended carrier testing for all informative family members to recognize mutations in asymptomatic family members.

## Introduction

Beta-ketothiolase deficiency also known as alpha-methyl acetoacetic aciduria, T2 deficiency, mitochondrial acetoacetyl-CoA thiolase deficiency, 3-oxothiolase deficiency and 2-methyl-3-hydroxybutyric acidemia (1-4).

**Fig. 1:**
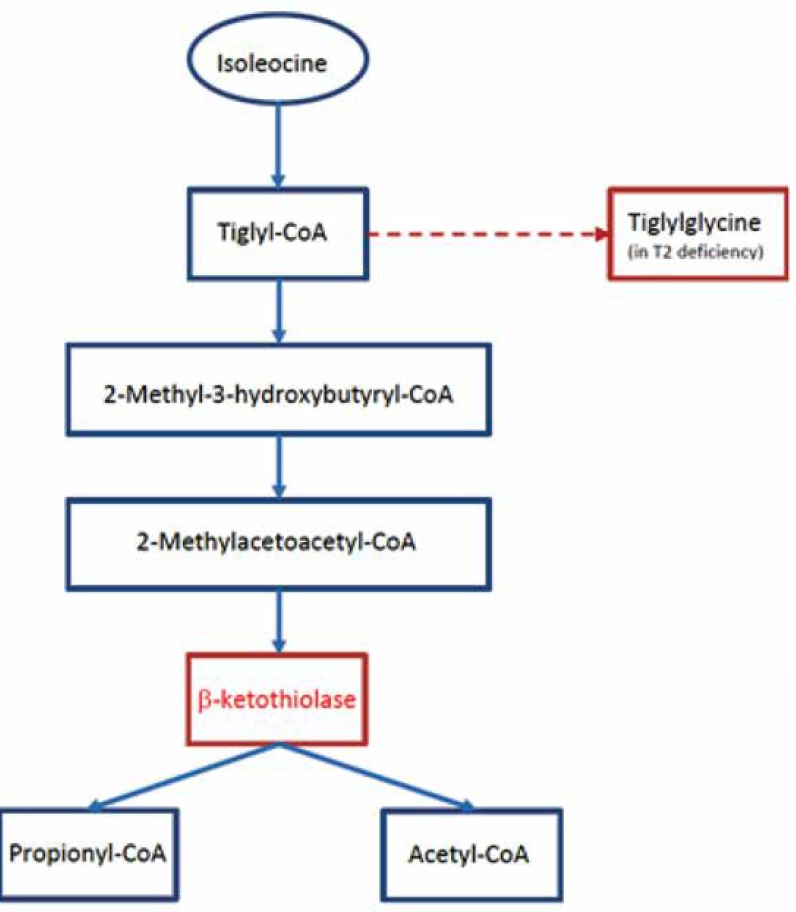
Isoleucine metabolism and metabolic block in T2 (ketothiolase) deficiency (5).

The T2 deficiency (as an abbreviation of beta-kethothiolase deficiency) is an autosomal recessive organic aciduria due to the deficiency of mitochondrial beta ketothiolase enzyme affected in pathway of ketone body metabolism and isoleucine catabolism (Figure 1) (5, 6). 

Currently, more than 100 patients have been reported worldwide with no local predisposition (1). This rare condition is estimated with an incidence of 1 per 1 million newborns or less (2,7).

This disorder first described in 1971 in 6 years old boy. The presenting clinical feature was severe episodes of metabolic acidosis with increased amounts of alpha-methyl beta- hydroxyl butyric acid in the urine (8).

The typical clinical features manifest between age of 6 to 18 months in infancy with intermittent attacks of ketoacidosis crisis, vomiting, hypotonia, respiratory distress, convulsion, and lethargy. Coma, psychomotor retraction and even death have been reported in rare cases (9). These acute crises can decompensate rapidly in early childhood and lead to neurodevelopmental delay, repeated seizures and death without appropriate treatment (10).

The episodes often brought on by stress, fasting, acute illness, fever and infection (2). The frequency of episodes decreases with age and between episodes, patients are often asymptomatic. Severity of disease varies among patients but outcome can be good (11).

In rare cases, atypical presentation can happen with having sign and symptoms before age of 6 months, even in neonatal period (12), also some patients can present with neurological manifestations before a first ketoacidotic crises, however, is very rare (13).

This disorder can be suspected with presence of keto acidotic attacks, increased urinary level of 2-methyl-3-hydroxybutyrate, 2-methyl acetoacetate and tiglylglycine witch revealed by urinary organic acid analysis and/or increased levels of tiglycarnitine and 2-methyl-3-hydroxybutylcarnitine in blood plasma analysis (1, 3). Quantitative organic acid analysis can use for any patient presenting with attacks of unexplained ketoacidosis and clinical symptoms of hypotonia, seizure, motor or mental delay of unknown cause (2, 3). According to clinical phenotype and distinctive laboratory finding, genetic assay can help for confirming the diagnosis by ACAT1 gene mutation analysis (14).

Early diagnosis of T2 deficiency especially in asymptomatic cases and appropriate management will prevent its serious complications and improve final prognosis (2).

## Case Presentation

A two months old Iranian girl born to consanguineous parents (cousin), presented in our center (Imam Reza Hospital, Mashhad, Iran) in October 2016 for evaluation of seizure and hypotonia in Pediatric Department. She had one sibling who died in 7 months old with similar symptoms and no more specific metabolic assessment.

Her mother had pregnancy-induced hypertension. She was a full term baby with birth weight of 2500 gr, birth length of 45 cm, birth head circumference of 32 cm and normal APGAR score.

She admitted at hospital in the fifth day of life with chief complaint of neonatal jaundice. In her first presentation at 2 months old, she had a history of fever, poor feeding and vomiting for 2d after routine vaccination. Her condition deteriorated with tonic-clonic seizures, difficulties in breathing, severe restlessness, lethargy, hypotonia and come for 1 day in her admission in intensive care unit. 

After first admission, she had four more episodes with similar signs and symptoms and between these crises; she was asymptomatic. Developmentally, at 8 months, she could not babble, have head drop. She could not sit with support. Physical examination revealed no organomegaly. Not hearing or visual abnormalities.

Neurological examination results showed hypotonia and decreased deep tendon reflexes.

Arterial Blood Gas test revealed metabolic acidosis with PH: 7.2, HCO3: 5mmol/L, base excess: -20, and pCO2: 12.5 mmHg in her first acute attack. High level of blood sugar and ketonuria was detected. The patient’s plasma showed normal Ammoniae (72 µmol/L) and lactate (11 mg/dl). Beside negative Urine and blood culture, serum electrolytes, liver and renal and thyroid function tests had normal results. CSF analysis revealed negative results for infection.

**Fig. 2:**
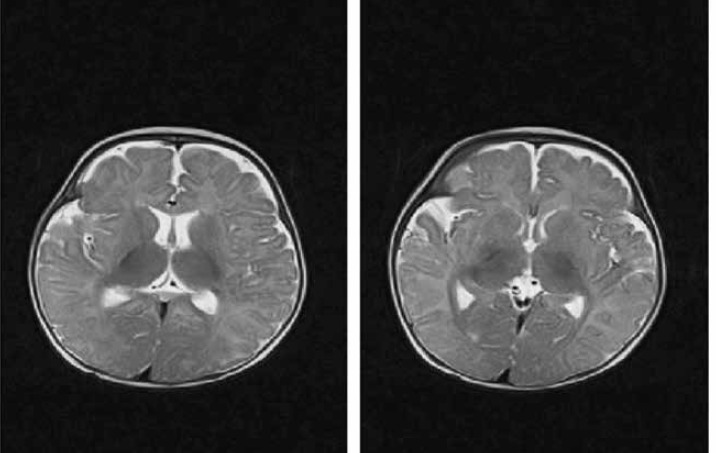
Brain Magnetic Resonance Imaging shows nonspecific low signal intensity in basal ganglia

Brain Magnetic Resonance Imaging (MRI) showed unspecific low signal intensity basal ganglia (Figure 2). Tandem mass spectrometry (MS/MS) showed hydroxy methylglutaryl CoA-lyase deficiency and 3- methyl coronyl– coalyase deficiency. Suggested diagnosis was beta keto-thiolase deficiency (oxothiolase deficiency).

The organic acids in her urine showed an elevated 2methyl -3hydroxybutiric acid, an elevated tiglyglycine and 4 – hydroxy phenyl lactic acid.

**Table 1:** Genetic assay relevance to patient’s phenotype

Gene (transcript)	Nucleotide (protein)	Zygosity	Described by	Disorder (OMIM#, inheritance
ACAT1 (NM_000019.3)	c.664A>C (p.Ser222Arg)	Hom.	Not described	Alpha-methylacetoacetic aciduria (203750, AR)

**Table 2:** Gene mutation analysis in beta-ketothiolase deficiency in Asian and Arab population

**Population**	**Study type**	**ACAT1 mutation analysis**
Iran	Case report (novel)	c.664A>C (p.Ser222Arg)
United Arab Emirates	NBS program retrospective	c.86-87 dupTG c.854C>T
Vietnam	Case series	p.Arg208
India	Case report	c.578T>G
Lebanese origin	Case report (novel)	c.1124A>G
Libya	Case report	c.674C>A
China	Case series	c.653C>T (p.S218F)
Japan	Case report	c.951C>T c431A>C

Patient referred to gene assay diagnosis by whole exome sequencing examination. Approximately 37 MB (214,405 exons) of the Consensus Coding Sequences (CCS) were enriched from fragmented genomic DNA and were evaluated by Centogene AG Department. In genetic assay, we found a novel homozygous mutation c.664A>C (p. ser 222Arg) in ACAT1 gene that was the first time we detect this variant (Table 1). 

These results were consistent with a genetic diagnosis of beta-ketothiolase deficiency (alpha-methyl acetoacetic aciduria) in our patient. 

## Discussion

Beta ketothiolase deficiency is a rare disease that affects isoleucine catabolism and ketone body utilization. The common age of onset is between 6 and 24 months. It is clinically characterized by acute attacks of ketoacidosis with vomiting, dehydration, lethargy, and seizure that can trigger with infection, stress and fasting (2, 5). T2 deficiency can be considered in the differential diagnosis for ketoacidosis in children without marked reason (15).

In this case report, we described a 2-month old girl with Beta-ketothiolase deficiency who initially presented with seizure, hypotonia, and hyporeflexia. In other reports, the first presentation varies from neonatal age to childhood. In Libya, there is a report of 2 siblings with T2 deficiency with different first presentation in 7 months age and 4 yr old age, that shows the various age presentation even in a family (3,15).

Patients commonly have clinical presentation of metabolic encephalopathy like vomiting, seizures, and loss of consciousness(15) which could astound the correct diagnosis.

As mentioned this disorder can be suspected with presence of keto acidotic attacks, increased urinary level of 2-methyl-3-hydroxybutyrate,2-methyl acetoacetate and tiglylglycine witch revealed by urinary organic acid analysis and/or increased levels of tiglycarnitine and 2-methyl-3-hydroxybutylcarnitine in blood plasma analysis (1, 3).

This disorder can be diagnosed in prenatal period, but it can manifest rarely during neonatal period (3). New born screening programs (NBS programs) can detect beta-ketothiolase deficiency in first few days of life, before appearance of clinical features; however, some developing countries may not have this routine screening (2). In addition, some patients cannot be detected by metabolic screening, so true incidence of beta ketothilase deficiency is uncertain. More than 100 patients have been reported from different countries (1). The first report of our country was a 6 month-old Iranian boy presented with clinical symptoms and diagnostic urinary organic acid analysis for beta -ketothiolase deficiency. Genetic assay was not reported for this case (16). We do not have clear incidence in Iran, but in three Middle East countries to be 1.4, 1.6 and 2.76 per 100000 newborn in Qatar, Saudi Arabia and United Arab Emirates (17-19).

T2 deficiency can be confirmed by mutation in ACAT1 gene in DNA genomic analysis (2). First time, 2 mutations in ACAT1 gene were found, that it was the first definition of a mutant ACAT allele and the diagnosis of beta ketothiolase deficiency was made by urinary organic acid analysis (20). ACAT gene is located on chromosome 11q 22.3 to q23.1 is 27-kb in length and contains 12 exons and 11 introns (20). Today there are more than 70 different mutations resulting beta ketothiolase deficiency in different population and few novel mutations have been reported worldwide (2, 15). In a descriptive study of 35 patients with T2 deficiency in Vietnam, the most common mutation of ACAT1 gene was R208X (p.Arg208) in 70% of cases, that it is the most common mutation has been identified in the world (21). Except this mutation in Vietnamese (p.Arg208), no other common ACAT1 mutations have been reported (15). In Asian and Arab countries, there are some reports of ACAT1 mutations and novel mutations (Table.2) (17, 22-26). In our study, we found a homozygous mutation c.664A>C (p. ser 222Arg) in ACAT1 gene by whole exome sequencing test, that was a novel mutation, so it has not been reported before.

Today, the DNA analysis of suspected gene in patients with beta ketothiolase deficiency can confirm this disorder. In gene, analysis reveal novel variants in ACAT1 gene that have never been reported, can be enough for diagnosis of beta-ketothiolase deficiency (7, 26). Furthermore, in 2-methyl 3hydroxybutyryl- COA dehydrogenase deficiency, the biochemical profile may mimic the beta -ketothiolase deficiency disorder, so a genetic analysis of ACAT1 gene can help to confirm the diagnosis (15). In patients with beta-ketothiolase deficiency, early diagnoses and proper management can make a favorable outcome. In addition, screening of other family members with genetic counseling can identify asymptomatic members. In addition, a prenatal diagnosis can be useful consideration in early diagnosis and management in patients, but clinical manifestations in neonatal period are rare (3). Recurrent attacks of ketoacidosis, biochemical findings, and mutation analysis of ACAT1 gene, the diagnosis of beta-ketothiolase deficiency correlated with our patient. With the proper management, our patient is in a good condition without any acute episode in recent 2 months.

**In conclusion, **in patients with beta-ketothiolase deficiency early diagnose and proper management can make a favorable outcome. In addition, screening of other family members with genetic counseling can provide the asymptomatic members. Moreover, a prenatal diagnosis can be useful consideration in early diagnosis and management.

Because of the vast clinical spectrum of beta-ketothiolase deficiency, and preventive treatment can decrease or omit the incident of metabolic crises, our suggestion would be screening of patient’s siblings even if they are asymptomatic and have no history of keto acidotic crises.

On the other hand, challenge of subtle abnormalities in urinary organic acid profile and the necessity of early exact diagnosis emphasis on using genetic assay or an enzymatic analysis to confirm the diagnosis.
